# Prostate Cancer Associated Lipid Signatures in Serum Studied by ESI-Tandem Mass Spectrometryas Potential New Biomarkers

**DOI:** 10.1371/journal.pone.0150253

**Published:** 2016-03-09

**Authors:** Divya Duscharla, Sudarshana Reddy Bhumireddy, Sridhar Lakshetti, Heike Pospisil, P. V. L. N. Murthy, Reinhard Walther, Prabhakar Sripadi, Ramesh Ummanni

**Affiliations:** 1 Center for Chemical Biology, CSIR-Indian Institute of Chemical Technology (CSIR-IICT), Hyderabad, India; 2 Centre for Academy of Scientific & Innovative Research, CSIR-Indian Institute of Chemical Technology (CSIR-IICT), Hyderabad, India; 3 National Centre for Mass Spectrometry, CSIR-Indian Institute of Chemical Technology (CSIR-IICT), Hyderabad, India; 4 High Performance Computing in Life Sciences, Technical University, Wildau, Germany; 5 Department of Urology, Nizam’s Institute of Medical Sciences (NIMS), Hyderabad, India; 6 Department of Medical Biochemistry and Molecular Biology, University of Greifswald, Greifswald, Germany; Hormel Institute, University of Minnesota, UNITED STATES

## Abstract

Prostate cancer (PCa) is one amongst the most common cancersin western men. Incidence rate ofPCa is on the rise worldwide. The present study deals with theserum lipidome profiling of patients diagnosed with PCa to identify potential new biomarkers. We employed ESI-MS/MS and GC-MS for identification of significantly altered lipids in cancer patient’s serum compared to controls. Lipidomic data revealed 24 lipids are significantly altered in cancer patinet’s serum (n = 18) compared to normal (n = 18) with no history of PCa. By using hierarchical clustering and principal component analysis (PCA) we could clearly separate cancer patients from control group. Correlation and partition analysis along with Formal Concept Analysis (FCA) have identified that PC (39:6) and FA (22:3) could classify samples with higher certainty. Both the lipids, PC (39:6) and FA (22:3) could influence the cataloging of patients with 100% sensitivity (all 18 control samples are classified correctly) and 77.7% specificity (of 18 tumor samples 4 samples are misclassified) with *p*-value of 1.612×10^−6^ in Fischer’s exact test. Further, we performed GC-MS to denote fatty acids altered in PCa patients and found that alpha-linolenic acid (ALA) levels are altered in PCa. We also performed an *in vitro* proliferation assay to determine the effect of ALA in survival of classical human PCa cell lines LNCaP and PC3. We hereby report that the altered lipids PC (39:6) and FA (22:3) offer a new set of biomarkers in addition to the existing diagnostic tests that could significantly improve sensitivity and specificity in PCa diagnosis.

## Introduction

Despite the significant advances achieved in diagnosis and treatment of PCa, still it is the second most cause of cancer-related deaths,next to lung cancer among men [1]. Autopsy investigations have revealed that 30% of men above 50 years and 80% of men above 70 years have evidence for occurrence PCa [2]. Early diagnosis and aggressive treatment is the only option to cure PCa. Biomarkers play an important and decisive role in early diagnosis of PCa. Till date, screening of for PCa involves the digital rectal examination (DRE) and the prostate specific antigen (PSA) blood test. However, these two tests currently in use for PCa diagnosis are sub-optimal because PSA is abundantly produced by prostatic epithelium and also secreted by the epithelium of periurethral glands. PSA expression is not specific to tissue or gender, it will be secreted by both benign and cancer cells of prostate [3]. Advanced “Omics” technologies have identified altered genome, transcriptome and proteome related to PCa. These studies have provided a number of potential genomic and proteomic biomarkers for diagnostic purposes. However, none of these markers are translated into routine diagnostic and/or prognostic applications. Consequently, there are innumerable chances for either over diagnosis of patients with limited potential for cancer or under diagnosis of patients already suffering from the disease. Thus lack of current diagnostic methods for prostate diseases underscores the need for improvement in this area.

Diagnosing cancers based on serum profiling is an attractive concept. A number of studies have employed proteomic and genomic analysis of serum samples from cancer patients. Only sparse information is available on metabolome alterations particularly lipid composition in serum/plasma associated with PCa. Abnormal lipid metabolism has been shown to be associated with many diseases such as inflammation, diabetes, renal and heart failures as well as many cancers; thus indicating that lipid metabolites could be used as disease biomarkers [4]. Unfortunately, to the best of our knowledge, only a few reports have been found analyzing lipids from PCa patients associated with disease progression. One study reported that apolipoprotein and cholesterol together diagnosed ovarian cancer with 97% accuracy [5]. In colorectal cancer, altered linoleic acid (LA), alpha-linolenic acid (ALA), arachidonic and oleic acids were shown to be associated with cancer progression [6]. However, in these studies only a few classes of lipidswere analyzed due to the technical limitations. Two recent studies have reported that thephospholipidalterations are associated with PCa [7,8]. Particularly cancer progression depends on the proliferation and invasion of tumor cells to distal organs. Tumor cells undergo functional and morphological alterations, many of which start with cell membrane releasing their components into blood stream. Lipidomicsapproach has been applied to identify biomarkers and study role of lipids in disease progression of many metabolic disorders such asobesity [9], atherosclerosis [10], hypertension [11], diabetes [12], cystic fibrosis [13] and cancers [14]. Recently Patel et al. have reported that a three lipid signature (phospholipids) can distinguish PCa patients from normal individuals [8]. Therefore, we sought to investigate complete lipid profiles covering various classes of lipids including fatty acids, TGs, DGs and phospholipids in serum from PCa patients. These lipid signatures might be used for screening of PCa patients along with existing diagnostic tests with better specificity and sensitivity. Furthermore, we believe that lipidomicapproach will identify lipids and their associated pathways, which may play a role in PCa initiationand progression.

## Materials and Methods

### Chemicals

Internal standards (heptadeconoic acid and methyl heptadecanoate) as well as saturated and unsaturated fatty acids used in the study were purchased from Sigma-Aldrich (St. Louis, MO, U.S.A). Analytical grade solvents were used to prepare the stock solutions of fatty acids. Ethanol was purchased from Commercial Alcohols, Canada. Methanol used in the ESI-MS analyses was purchased from MERCK, Mumbai, India. Diazomethane in ether is used for esterification (methylation) of fatty acids by known method.

### Clinical Samples and Ethics Statement

The serum samples and subsequent pathological data were collected after obtaining patient’s consent by filling the consent forms which were prepared and approved by the institutional ethics committee. The institutional ethics and bio safety committee of the Nizam’s Institute of Medical Sciences (NIMS) Hospital, Hyderabad, India approved the present study. For lipidomics, blood samples were collected from patients with high serum PSA values and pathological examination prior to any chemotherapy or surgery. Serum samples separated from whole blood were stored at -80°C until total lipids were isolated using solvent extraction procedure.

### Selection of patients and sample collection

For the present study serum samples were collected from 18 patients with elevated PSA levels diagnosed with PCa from NIMS hospital sample archive for research purposes. All patients selected for this study did not undergo any treatment and/or surgery before collecting the blood samples. For confirmation of PCa, the diagnosis for each patient was established by histopathology of prostate biopsies. Each patient’s information including their age, serum PSA value and pathological diagnosis such as tumor grade and Gleason scoreis provided in Table 1. For the control group, 18 serum samples from male controls were obtained from institute dispensary where patients had their routine check for wellness or for diagnosis of other diseases. We had set a criteria including age matching with no history of the diagnosis of PCa for collecting these control samples. The serum samples were collected from both tumor and control group individuals in the same manner. From each individual, 5 ml of whole blood was collected in a vacutainer tube containing Trisodium citrate. The tubes were allowed to stand for 10 min for clotting and then centrifuged at 3000 RPM to collect serum. The clear supernatant from surface was collected and stored as separate aliquots at -80°C until use.

**Table 1:** List of patients diagnosed with PCa included in the study. The clinical parameters of tumors Gleason score and PSA values are provided.

S.No	ID	Pre/Post	Age	Weight	PSA	Gleason Score	Metastasis
1	**Tumor-1**	Pre	60	58	100	3+3 = 6	No
2	**Tumor-2**	Pre	75	47	100	4+5 = 9	Yes
3	**Tumor-3**	Pre	65	70	31.2	4a+4b = 8	Yes
4	**Tumor-4**	Pre	60	50	89.5	3+4 = 7	No
5	**Tumor-5**	Pre	65	72	32	3+3 = 6	No
6	**Tumor-6**	Pre	75	70	114.6	3+4 = 7	Yes
7	**Tumor-7**	Pre	75	75	13.2	3+4 = 7	No
8	**Tumor-8**	Pre	68	75	44.1	3+3 = 6	No
9	**Tumor-9**	Pre	55	65	155.4	3+4 = 7	Yes
10	**Tumor-10**	Pre	61	72	0.1	5+4 = 9	No
11	**Tumor-11**	Pre	78	50	6	4+3 = 7	No
12	**Tumor-12**	Pre	NA	NA	6	3+3 = 6	No
13	**Tumor-13**	Pre	NA	NA	20	3+4 = 7	No
14	**Tumor-14**	Pre	64	NA	28	5+4 = 9	YES
15	**Tumor-15**	Pre	NA	NA	8	NA	No
16	**Tumor-16**	Pre	64	NA	12	5+4 = 9	YES
17	**Tumor-17**	Pre	60	75	0.01	5+4 = 9	No
18	**Tumor-18**	Pre	NA	56	21	3+3 = 6	No

### Isolation/extraction of total lipids from serum samples

Total lipids from prostate cancer and normal serum samples were extracted as reported previously by Bligh and Dyerwith minor modifications [15]. Briefly, lipids were extracted from serum samples by substituting chloroform with dichloromethane (DCM)[16,17]. A 30 μl aliquot of serum was spiked withan internal standard (1.5 μl of 50 μM methyl heptadecanoate for positive ion ESI-MS analysis or heptadeconoic acid for negative ion ESI-MS analysis), and added with 190 μl of MeOH. The mixturewasvortexed for 30 Sec and then 380 μl of DCM was added to the mixture and again vortexed for 30 Sec. Finally, to enhance the separation efficiency of two phases, 120 μl of water was added and mixed by vortexing for 15 Sec. Then the mixture was allowed to equilibrate at room temperature for 10 min and subjected to centrifugation at 8000g for 10 min at 10°C for separation of phases. The upper layer was carefully removed. The lower lipid-rich DCM layer was collected into a separate 1.5 ml micro tubeandthe solvent was evaporated in centrivap under vacuum at 4°C. Finally, the dried lipid extracts were reconstituted in 100 μl of buffer (ACN/IPA/H_2_O in 65:30:5 v/v/v) before subjecting to direct ESI-MS analysis. For GC-MS analysis the dried lipid extract were further subjected to methylation using diazomethane.

### Electrospray Ionization Mass Spectrometry (ESI-MS) analysis

The experiments were performed using a quadrupole time-of-flight mass spectrometer (QSTAR XL, Applied Biosystems/MDS Sciex, Foster City, CA, USA) equipped with an ESI source, acquiring data using Analyst QS software (Applied Biosystems). All the samples were introduced into the source by flow injection (10μl loop) using methanol as the mobile phase at a flow rate of 0.03 ml/min. The samples were analyzed under positive and negative ESI conditions. The typical positive ion ESI conditions were: capillary voltage, +5 kV; declustering potentials (DP1), 60 V; DP2, 10 V; focusing potential, 250 V. The typical negative ion ESI conditions were: capillary voltage, -4.5 kV; DP1, 60 V; DP2, 10 V; focusing potential, 250 V. Full scan mass spectra were recorded over the mass range of *m/z* 50–2000 using a time-of flight (TOF) analyzer at a resolution of 10,000 Full Width Half Maximum. Nitrogen was used as the curtain gas and the collision gas, whereas air was used as the nebulizer. For structure identification, collision-induced dissociation (CID) spectra were recorded by selecting the precursor ion of interest using the quadrupole, allowing them to fragment in the collision cell, and separating the product ions by the TOF analyzer. The collision energies used were between 5 to 25 eV. The experimental conditions used for samples are as same as for reference standards. All the spectra reported were averages of 25 to 30 scans. Elemental compositions of all the precursor ions as well as product ions were obtained from the accurate mass values using the Analyst software.

### GC-EIMS analysis

GC-EIMS analyses were performed using an Agilent 6890 gas chromatograph (Agilent Technologies, Palo Alto, CA) equipped with a 5973N mass selective detector (MSD). HP-5MS capillary column (30 m x 250 μm,i.d: 0.25 μm of film thickness) was used for the chromatographic separation of the fatty acid methyl ester (FAME) derivatives. 1 μl of the sample aliquot was injected into the GC-MS instrument in the splitless type of injection mode. The GC oven was programmed to increase from 50°C to 280°C at a rate of 10°C/min ramp and initial and final temperatures were held for 2 min and 5 min respectively. Total run time was 30 min. Helium was used as the carrier gas at a constant flow rate of 1 ml/min. The inlet and GC-MS interface temperatures were kept at 250°C and 280°C respectively. EI source and quadrupole analyzer were kept at 230°C and 150°C respectively. Data processing was done using MSD ChemStation software (Agilent Technologies, USA). The mass spectrometer was operated in the both full scan and selected ion monitoring (SIM) modes. The mass spectrometer was scanned from *m/z* 29 to 600 in full scan mode of analysis.

### Methylation of fatty acids for GC-EIMS analysis

Extraction method of total lipids from serum sample was same as discussed above except the last reconstitution step. For GC-MS analysis, dried serum lipid extracts were directly subjected to methylation using diazomethane reagent, by which the non-volatile fatty acids present in the serum samples were converted into volatile FAMEs. The sample vials containing dried lipid extracts were added with 1 ml of freshly prepared diazomethane in ether solution. The vials were quickly vortexed for 10 sec and left at room temperature for 10 min. The samples were concentrated to 50 μl using ScanVac. The standard fatty acids in ethanol (0.3 ml of 5 μM) subjected to methylation using the above mentioned procedure after evaporating ethanol. For identification of compounds, the same experimental conditions were maintained for the samples and reference standards.

### Data base search

The accurate mass values of all the detected peaks (*m/z*) in both positive and negative modes of ESI-MS analysis of extracts were taken into account, and they were searched in the metabolite databases with a mass tolerance of 5–10 ppm. The databases include Lipid Maps, Metlin (http://metlin.scripps.edu/index.php) and the Human Metabolome Database (HMDB) (http://www.hmdb.ca./spectra/ms/search) [18]. The database hits (with less ppm values) together with isotope distribution patterns were used for peak identification. Some of the critical metabolites were further confirmed by MS/MS analysis. The MS/MS spectra were compared with the data available in the literature as well as the American Oil Chemists Society (AOCS) lipid library (http://lipidlibrary.aocs.org/ms/ms16/index.htm) [7]. A few lipids were also confirmed from GC-MS data by comparing either the retention times of standards or the EI mass spectra available in AOCS lipid library.

### Statistical and bioinformatics analysis

The t-test was performed to compare the concentrations of identified lipid species between cancer patient and control (without PCa) groups. The lipid signatures showing differences with more than 1.5 fold increase or decrease with observed *p* value < 0.05were considered as differentially regulated lipids among PCa patients. Further an un-supervised analysis was performed to identify the best lipid signatures that could discriminate normal and tumor sample groups. Hierarchical clustering analysis (HCA), principal component analysis (PCA), correlation plots and partition analysis have been applied for the quantification data obtained. In addition, based on the abundance of lipids individually and/or in combinations, formal concept analysis (FCA) was used to predict classification of samples. FCA method is appropriate to find out significant relationships between altered lipids and clinical data of patients mathematically by graphical illustration[19]. These illustrations couldenvisagetheoretical hierarchy across the samples and data thus produced could be used to find out data dependency between sample attributes (clinical data and altered serum lipids). Recently, FCA is commonly used for theoretical clustering to identify combinatorial biomarkers and co-regulation of genes, proteins and metabolites [20,21]. In the present study, the FCA method was applied to identify any correlation between abundance of serum lipids and the classification (tumor and control groups) of samples. In FCA analysis theoretical lattices were built based on low or high abundance of lipids to understand the reliance of serum lipids abundance with PCa and normal samples. The lattices were drawn in combinations or single lipid species classified sample groups with different specificities.

### Cell proliferation assay

The human PCa cell lines LNCaP (androgen dependent cells) and PC3 (androgen independent cells) were obtained from ATCC and maintained in normal growth media (RPMI-1640 (Sigma) supplemented with 10% fetal bovine serum (FBS) along with 100 units/ml penicillin and streptomycin). Both the cell lines were cultured in humidified incubator at 37°C with a constant supply of 5% CO_2_. Mycoplasma contamination of cells in culture was controlled by regular testing using specific primers in RT-PCR. To determine the effect of alpha-linolenic acid on proliferation of PCa cell lines, LNCaP and PC3 cells were seeded in 12 well plates (1.0 X 10^6^ cells per well) in complete medium. Cells were allowed to grow for 24 h allowing them to attach on well surface. Then the cells were treated with indicated concentrations of ALA (0–25 μM) or vehicle control (solvent in which ALA is prepared). To measure cell count, cells were harvested at indicated time intervals and cell viability was estimated using countess (Invitrogen) in cell viability analyzer (Invitrogen). Both time dependent as well as concentration dependent effect of ALA on cell viability was determined in proliferation assays. The effect of ALA on cell morphology was determined by imaging the cells under microscope (Olympus Xi72, Japan).

## Results

The serum samples from normal (control) and cancer patients were processed identically as described in the experimental section. The sample aliquots were then subjected for direct high resolution ESI-MS analysis under positive and negative ion modes.

### Positive ion ESI-MS analysis

**Fig 1:**
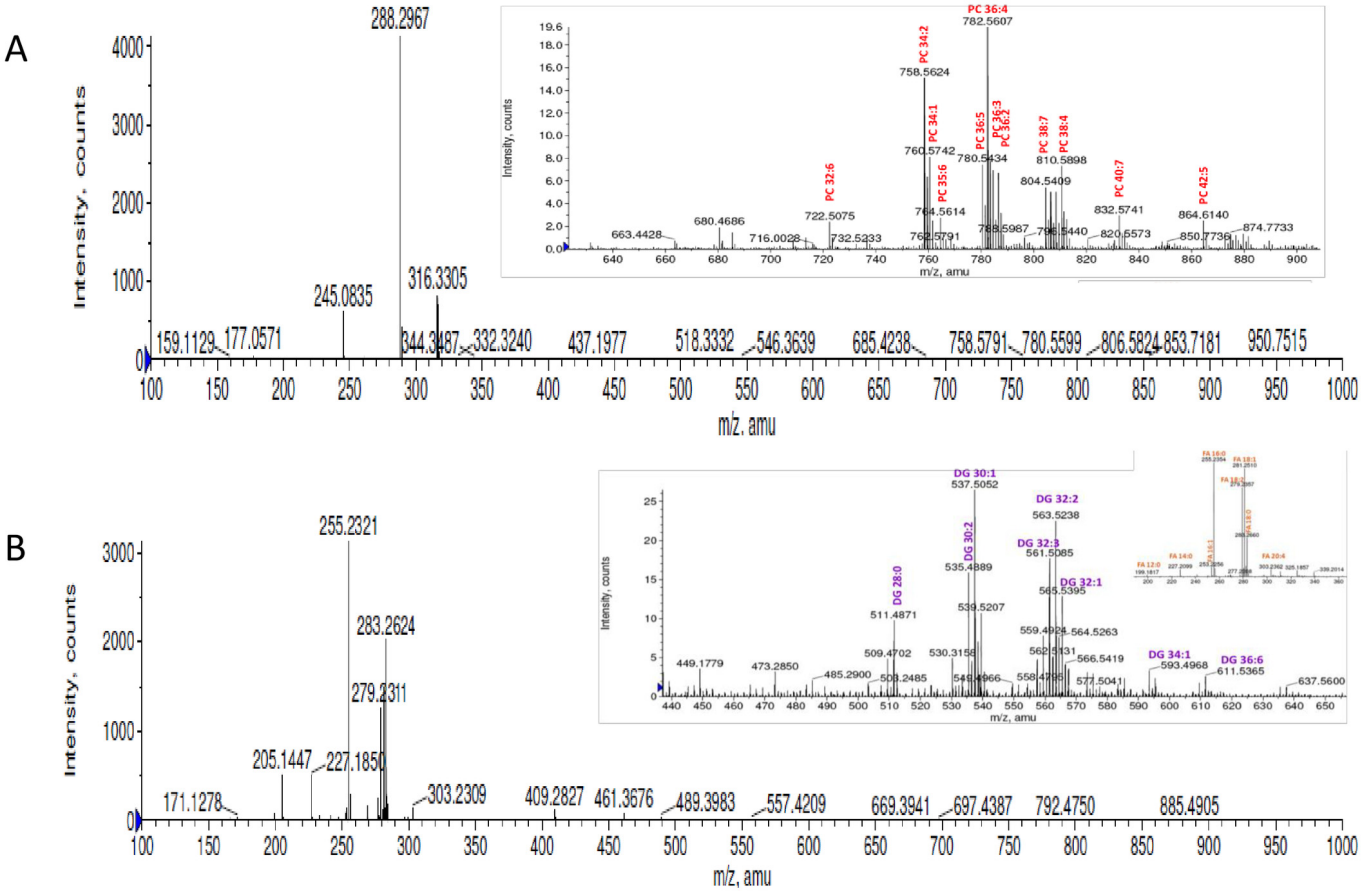
ESI mass spectrum of lipid extract of the serum sample from PCa patient recorded in a) Positive ion mode b) Negative ion mode.

The positive ion ESI mass spectra exhibited ions in the range of *m/z* 100–1000 due to various metabolites (Fig 1A). As the sample aliquots were analyzed by direct ESI-MS method, which avoids chromatography and desalting steps, the Na^+^ and K^+^ ions present in the serum remain in the sample aliquots. Thus, the detected ions could be protonated, sodiated and/or potassiated molecules, i.e., [M+H]^+^, [M+Na]^+^ and/or [M+K]^+^ ions, respectively. Hence, the detected ions in the positive ion mode were searched in the databases for all the possible ion species mentioned above.

**Fig 2:**
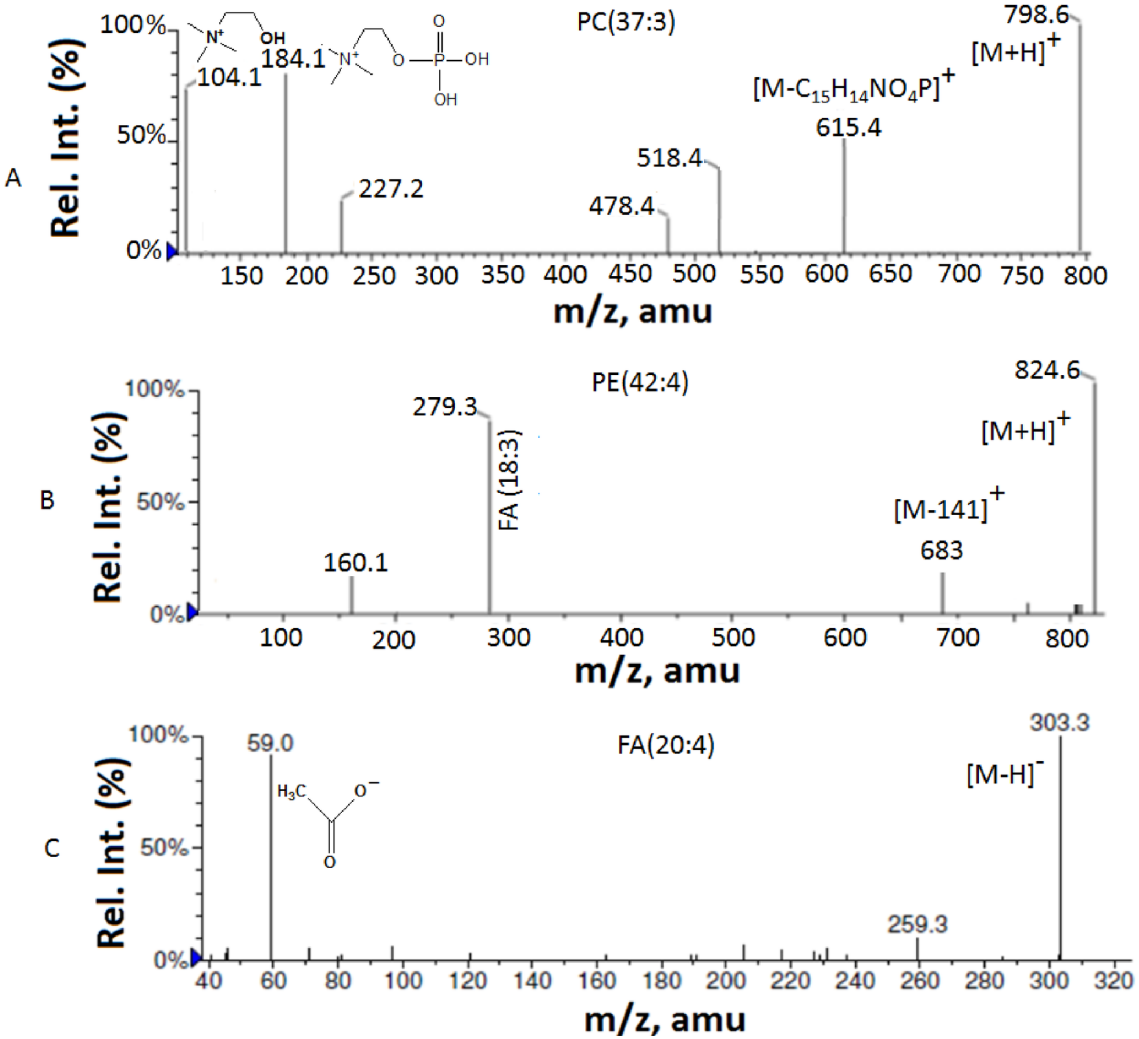
MS/MS spectra of a) [M+H]^+^ ion of PC(37:3), b) [M+H]^+^ ion of PE(42:4) and c) [M+H]^+^ ion of FA(20:4).

The database search results show that thedetected peaks in the ESI mass spectra include [M+H]^+^ ions of phosphatidylcholines (PCs), sphingomyelins (SMs), and phosphatidylethanolamine (PEs); [M+K]^+^ ions of fatty acids (FAs), diglycerides (DGs), triglycerides (TGs) and phosphatidic acid (PA); [M+Na]^+^ ions of DGs. Some of the key metabolites were confirmed by comparing their MS/MS data with that of corresponding standards available in the databases. The collision energy value used for the MS/MS experiments was 28 eV for PEs, 30 eV for PCs and 20 eV for SMs. Where the precursor ion intensities were lower, about 100–150 scans were combined (in MCA mode) to obtain their MS/MS spectra. The MS/MS spectra of the [M+H]^+^ ions of PC and PE and [M-H]- ion of FA are presented in Fig 2A–2C. The MS/MS spectra of [M+H]^+^ ions of phosphatidylcholine molecules generate the product ion at *m/z* 184 corresponding to its polar head group [22]. The ESI-MS/MS spectrum of PC (37:3) is shown in Fig 2A as an example. The spectrum showed the ion at *m/z* 184, the characteristic ion of the PC, and the ion at *m/z* 615 which provide the information about the acyl moieties attached to the glycerol. The ion at m/z 104 in the low mass region corresponds to the choline group. The MS/MS spectrum of PE (42:4) is shown in the Fig 2B, which showed the characteristic product ion due to the loss of 141 Da (ethanolaminephosphate) from the [M+H]^+^ ion, and this ion known to be specific for PE [23,24]. The other product ion in the spectrum appeared at *m/z* 279 provides the information about one of the acyl moiety of the PE as FA (18:3). The MS/MS spectrum of [M-H]^-^ ion of FA (20:4) is shown in Fig 2C. This spectrum matched well with the MS/MS spectrum of the standard FA (20:4) available in the Metline database.

### Negative ion ESI-MS analysis

The negative ion ESI mass spectra of lipid extracts showed [M-H]^-^ ions of different classes of lipid species (Fig 1B). Similar to positive ion ESI data, the accurate mass values of the detected peaks were searched against databases for the peak identifications. Based on database search results, the detected peaks in the spectra were found to be the [M-H]^-^ ions of fatty acids (FAs), DGs and Phosphatidic acid (PA). Some of the fatty acids were further confirmed by the MS/MS experiments on the [M-H]^-^ ions. The collision energies used were 25–30 eV for FAs. The MS/MS spectrum of FA 20:4 is shown in Fig 2D as a typical example and the spectrum showed characteristic product ions due to the loss of H_2_O (18 Da) and CO_2_ (44 Da) from [M-H]^-^ ions in addition to the product ion at *m/z* 59 corresponding to the acetate ion (CH_3_COO^-^) [25].

### Differential analysis & Quantification of lipids

The relative abundances of the peaks detected in control and patient samples were normalized based on the internal standard peak intensities. The normalized values were subjected for determining differentially regulated lipid signatures with minimum of 1.5 fold increase or decrease in cancer patients compared to control group. ANOVA test was performed for statistical significance (< 0.05) of altered lipids across the PCa patients. HRMS data of the differentially regulated lipid species with corresponding fold differences are summarized in Table 2. All identified lipids were classified into seven different classes PC, PA, PE, TG, DG, SPL and FA. Except fatty acids, remaining six classes of lipids were higher in patient serum samples than those of control samples. Among the individual classes, TGs were elevated in PCa patients compared to normal healthy individuals.

**Table 2:** High resolution mass spectral data. The identified metabolites in the lipid extracts of the serum samples.

S.No	Lipid species	Molecular formula	Ion	Theoretical *m/z*	Observed *m/z*	Error (ppm)	Fold change (P/C)	Regulation
**Phospolipids**								
1	PA(14:0)	C_17_H_35_O_7_P	[M-H]^-^	381.2048	381.2066	-4.72	0.67	Down
2	PA(32:1)	C_35_H_67_O_8_P	[M+K]^+^	685.4205	685.4238	-4.81	4.09	UP
3	PC(37:3)	C_45_H_85_NO_8_P	[M+H]^+^	798.6007	798.5964	5.3	2.08	UP
4	PC(39:6)	C_47_H_82_NO_8_P	[M+H]^+^	820.5851	820.5815	4.38	1.99	UP
5	PE(29:1)	C_34_H_66_NO_8_P	[M+K]^+^	686.4158	686.4191	-4.8	7.8	UP
6	PE(31:3)	C_36_H_66_NO_8_P	[M+H]^+^	672.4599	672.4642	-6.39	0.69	Down
7	PE(42:4)	C_47_H_86_NO_8_P	[M+H]^+^	824.6164	824.6108	6.79	4.19	UP
8	PE(42:5)	C_47_H_84_NO_8_P	[M+H]^+^	822.6007	822.5968	4.74	5.04	UP
**Fatty acids**								
9	FA(16:3)	C_16_H_26_O_2_	[M-H]^-^	249.1855	249.1846	3	0.69	Down
10	FA(18:3)	C_18_H_30_O_2_	[M-H]^-^	277.2168	277.2155	4.68	1.51	UP
11	FA(19:1)	C_19_H_36_O_2_	[M-H]^-^	295.2637	295.2621	5.43	2.06	UP
12	FA(20:2)	C_20_H_36_O_2_	[M-H]^-^	307.2643	307.2654	-3.58	0.66	Down
13	FA(20:4)	C_20_H_32_O_2_	[M-H]^-^	303.2324	303.2309	4.94	0.58	Down
14	FA(22:0)	C_22_H_44_O_2_	[M-H]^-^	339.3263	339.3248	4.43	0.28	Down
15	FA(22:3)	C_22_H_38_O_2_	[M+K]^+^	373.2509	373.2496	3.45	0.68	Down
**Di and Tri glycerides**								
16	DG(40:9)	C_43_H_66_O_5_	[M+K]^+^	701.4512	701.4487	3.56	4.18	UP
17	DG(30:2)	C_33_H_60_O_5_	[M-H]^-^	535.4368	535.4392	-4.48	0.61	Down
18	DG(32:3)	C_35_H_62_O_5_	[M-H]^-^	561.4525	561.4508	3.02	0.52	Down
19	DG(41:0)	C_44_H_85_O_5_	[M-H]^-^	693.6403	693.6355	6.92	1.5	UP
20	TG(52:3)	C_55_H_100_O_6_	[M+K]^+^	895.7152	895.7098	5.9	4.69	UP
21	TG(59:5)	C_62_H_110_O_6_	[M+H]^+^	951.8375	951.8322	5.56	4.38	UP
22	TG(60:11)	C_63_H_100_O_6_	[M+H]^+^	953.8532	953.8588	-4.08	4.43	UP
23	TG(61:5)	C_64_H_114_O_6_	[M+H]^+^	979.8688	979.8645	4.38	10.72	UP
24	TG(45:2)	C_48_H_88_O_6_	[M+K]^+^	799.6212	799.6164	6	2.26	UP

### Hierarchical clustering and partition analysis

**Fig 3:**
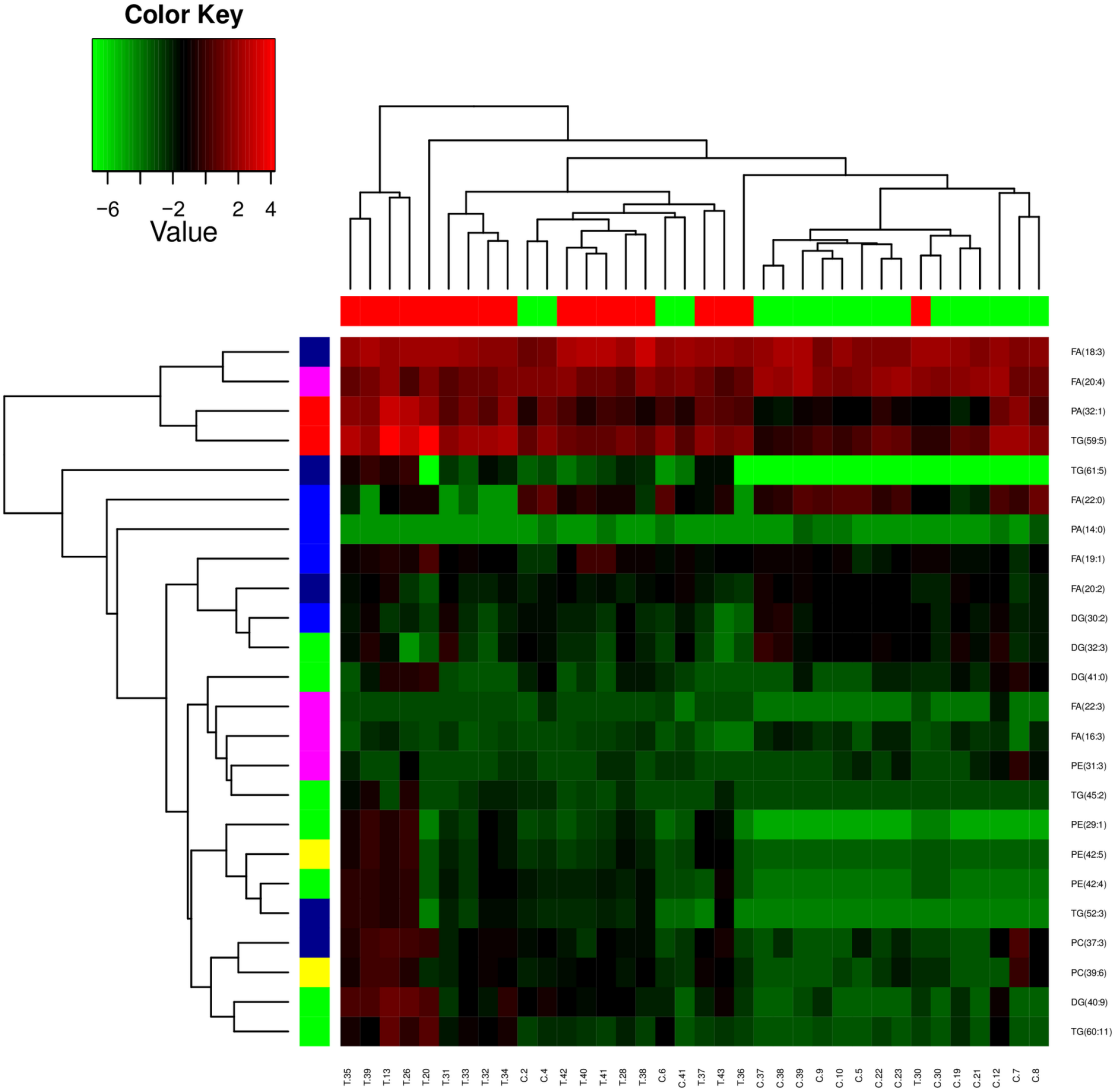
Unsupervised hierarchical clustering was performed using the log transformed values of lipid abundance determined from mass spectrometry data. On the heat map patients were shown horizontally whereas lipids vertically. In the upper color bar the cancer patients are marked in red, the controls are shown in green. In the side color bar each colour represents a specific class of lipids. The dendrograms represent the distance between the clusters.

**Fig 4:**
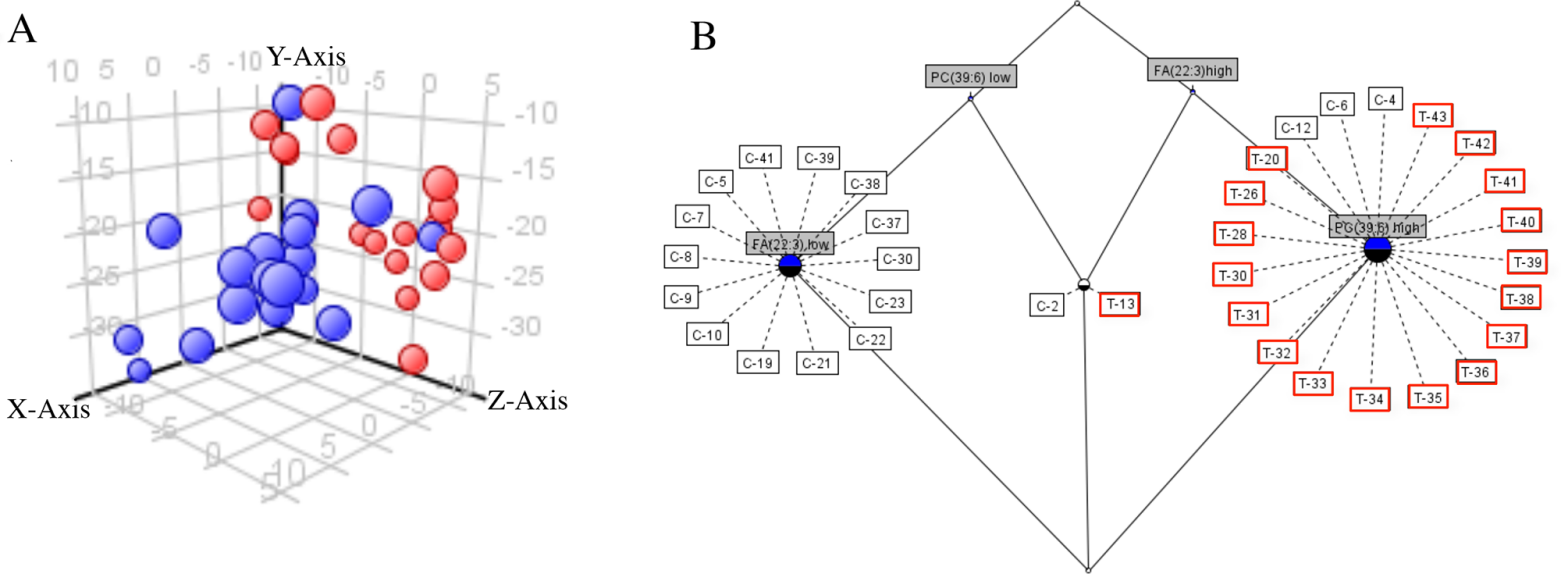
(A) Principal Component Analysis (PCA): Scatter plot of the top three principal components of PCA from the relative abundance of lipid data obtained from cancer and normal individuals. The blue balls indicatePCa patients, whereas red balls show non cancerous individuals. (B) Formal Concept Analysis to visualize sorting of samples based on the relative abundance of PC (39:6) and FC (22:3) in PCa (red squares) compared to control (black squares). This data dependant visualization has been performed to show partition efficiency of lipids individually and/or in combination to identify signature lipids of biomarker potential.

The hierarchical clustering of samples based on altered serum lipids is presented in Fig 3. On heat map, samples with higher expression are colored red while samples with lower expression are colored green. The columns represent samples and the rows indicate altered lipids in cancer patients serum compared to healthy individuals. The distances among the clusters are measured by dendrograms between the clusters. From the observed heat map two distinct clusters divide the tumor and normal samples with only minor exception. Nonetheless, hierarchical clustering revealed that 17 of 18 tumor samples (red) formed a discrete cluster. In case of controls, 14 samples (green) clustered together whereas the remaining four samples became part of the tumor samples cluster. In two-dimensional clustering, we also made an attempt to identify any specific lipid class forming a cluster that is mainly determining the tumor and control groups. The observed dendrograms confirm that no cluster is formed with single lipid class (FA, PE, PA, DG, PC and TG) (Fig 3). Based on the observed clusters, auxiliary PCA and partition analysis was performed. From the obtained relative abundance of lipids, a scatterplot with first three principal components shows a good partition between cancer patients and controls (Fig 4A). From the PCA analysis it is clearly visible that all tumors formed a unique component (blue), only one tumor sample became part of the control group component (red) based on 24 identified lipids. However, as reported by Lukk et al. only one individual differential biomarker between cancer and normal groups is not satisfactory to classify clinical samples with 100% specificity and sensitivity[26]. Therefore, partition analysis has been performed to determine potential lipid signatures for classification of samples into respective labeled group. Partition analysis of the relative abundance values highlighted two lipids, FA (22:3) and PC (39:6) which can classify samples with higher specificity (Fisher test with *p*-value 1.612e-06). Under this partition function if FA (22:3) is ≥ 0.045 ppm and PC (39:6) is ≥ 0.145 ppm, 100% sensitivity (18 control samples classified correctly PCa) and 77.7% specificity (of 18 tumor samples 4 samples are misclassified) could be achieved in classification of cancer patients from the corresponding normal group (Fig 4B). Partition analysis results also have indicated that the other identified lipids in combinations can classify all samples appropriately. From these results it is evident that more than one lipid of the same class or different class can distinguish tumor and control samples with higher specificity and sensitivity.

**Fig 5:**
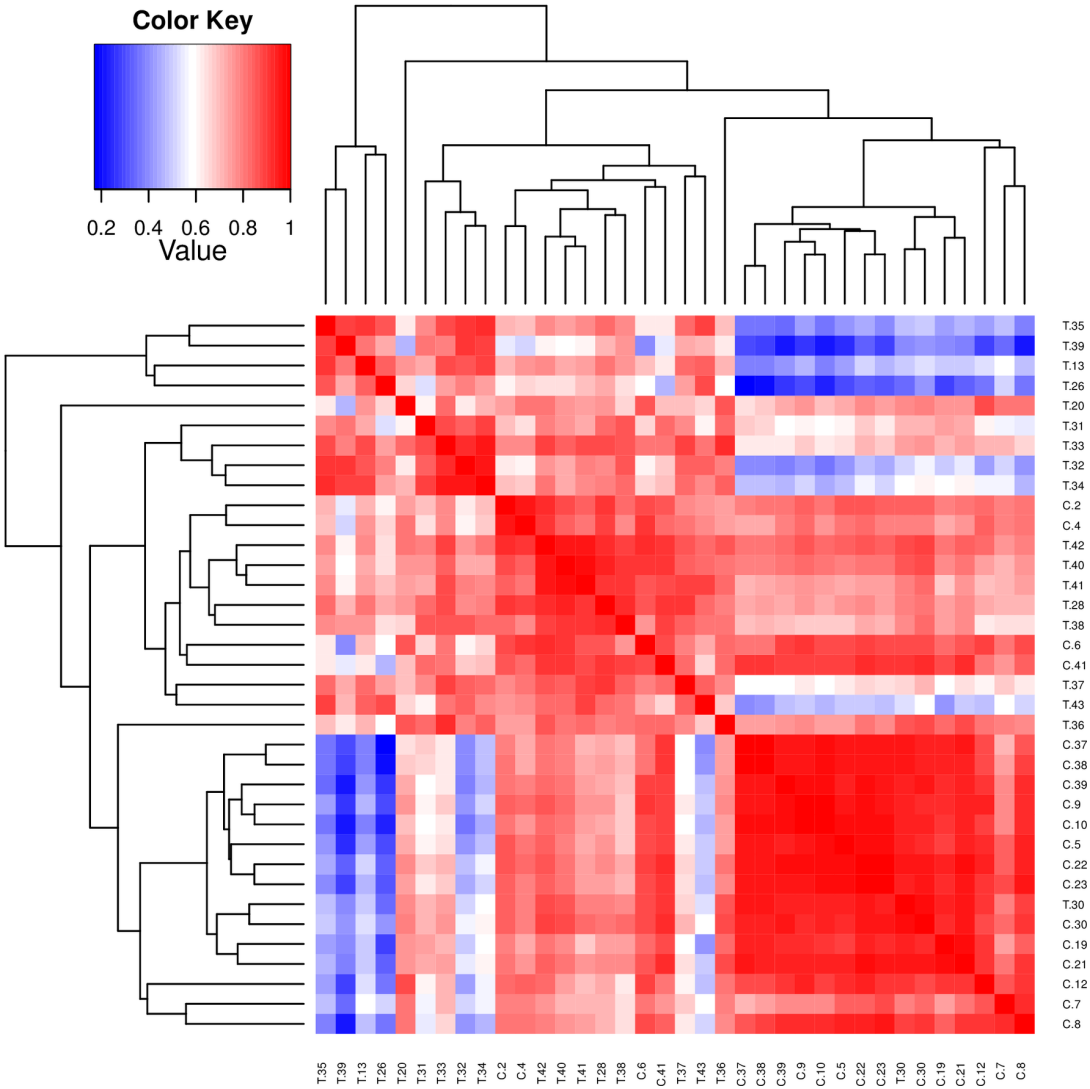
Sample-Sample correlation hear map for lipidomics data obtained. Clustering on correlation coefficients clearly demonstrates samples group (tumor or normal) based on the abundance levels of differential lipids.

**Fig 6:**
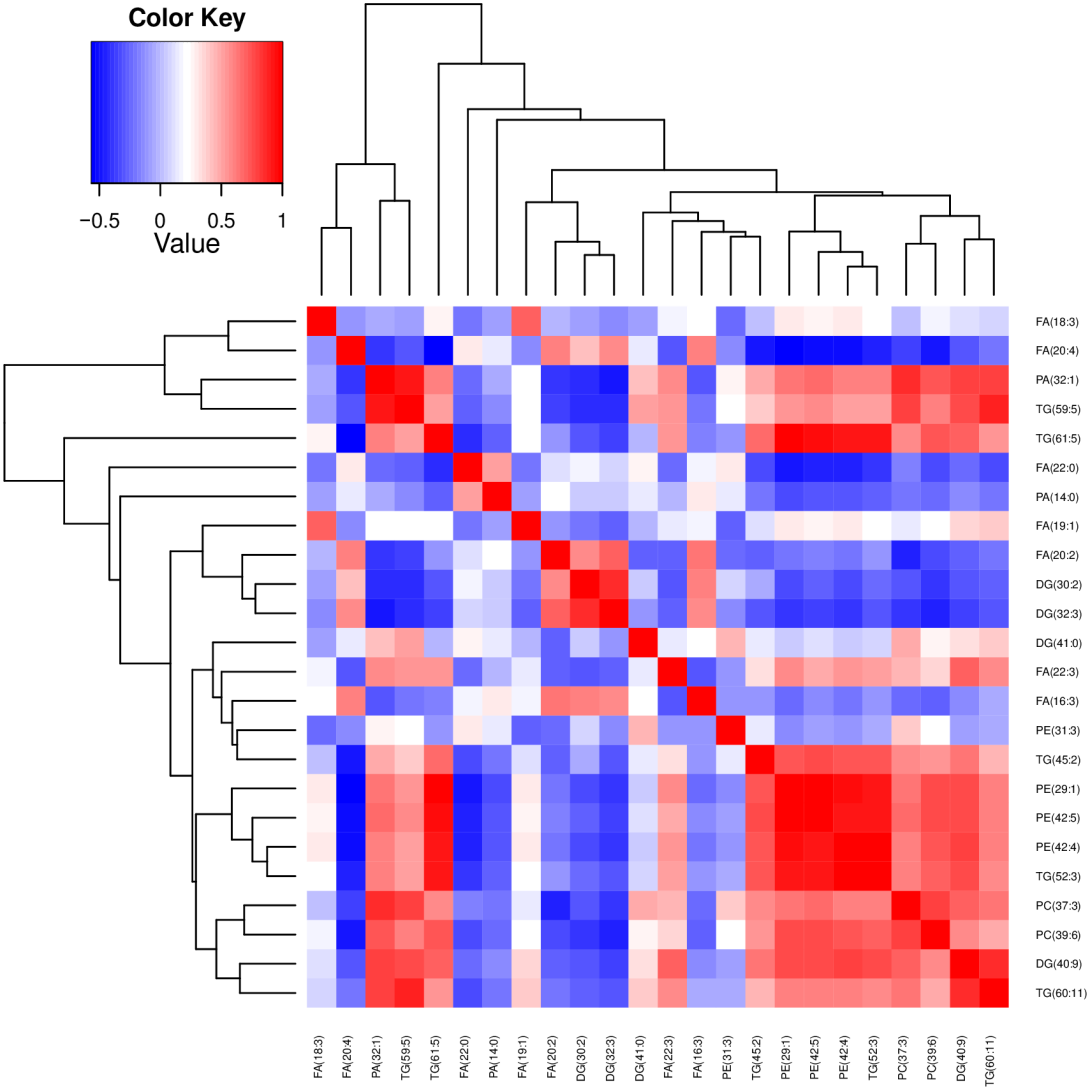
Heat map with pair-wise correlation calculation between entities (differential lipids identified from the current study)

To better understand the variation of lipids that altered in PCa patients, a correlation analysis was performed in a pair-wise manner to visualize dependency between abundance levels of identified lipids and serum samples. Correlation analysis allows identification of co-regulated lipids and identifies relationships between the samples within a study. The resulting correlation heat maps of log-transformed abundance levels of lipids using the Pearson correlation coefficients clearly differentiated major number of cancer patients with only one exception (Fig 5). In addition to sample-sample correlation, correlation between lipids determining their co-regulation has identified a major cluster of lipids containing 3 PEs, 2 PCs, 3 TGs and 1 DG. In particular both PCs and PEs clustered together showing good co-regulation of their serum abundance (Fig 6).

### Gas chromatography with electron ionization mass spectrometry (GC-EIMS) analyses

**Fig 7:**
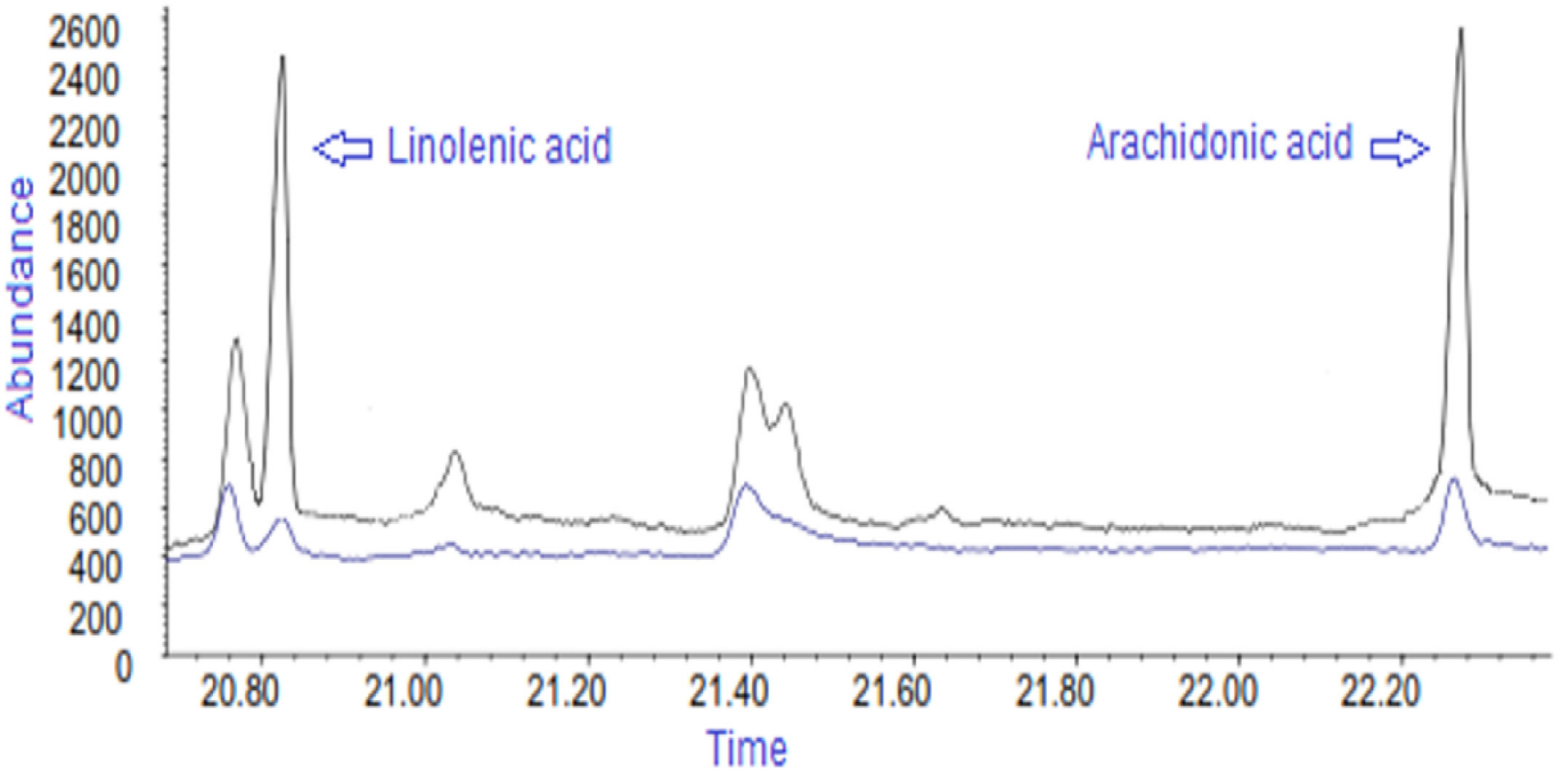
Representative GC-EIMS (SIM) chromatogram of a serums sample. Serum sample spiked with 4 μM of linolenic acid and arachidonic acid (black line)and serum samples analyzed without spiking fatty acids(blue line).

Fatty acids can be analyzed by GC-MS after derivatization (e.g., esterification, silylation etc.). In the present work, fatty acids were methylated by diazomethane and the FAMEs were subjected to GC-MS analyses under electron ionization (EI) conditions. The EI mass spectra of FAMEs are available in the AOCS lipid library (http://lipidlibrary.aocs.org/ms/ms16/index.htm). The spectra available include characteristic structure indicative fragment ions. The spectra are also available in commercial EI libraries (Wiley and NIST) as well as in the previously published reports [27]. Hence it is easy to identify the FAMEs in the GC-MS analysis by searching the target spectra against the EI library. We have used the EI library and/or standards to identify the fatty acids. In the present study, as expected, the saturated fatty acids showed predominantly two characteristic fragment ions at *m/z* 74 and 87 whereas polyunsaturated fatty acids showed the fragment ion at *m/z* 79 in the low mass region of the spectra. The EI spectra of methyl esters of heptadecanoic acid (17:0), linolenic acid (18:3) and arachidonic acid (20:4) are shown in S1A to S1B Fig. We could easily characterize high abundant fatty acids from their EI mass spectra (full scan mode analysis). In the case of low abundant fatty acids, we have performed GC-MS experiments in SIM mode by selecting specific fragment ions of target fatty acids. In this type of analysis, the retention parameters of target compounds are crucial. Consequently, before SIM experiments, the retention times of the target fatty acids were confirmed using the standards. By applying the GC-MS (SIM) method, we have confirmed presence of linolenic acid and arachidonic acid in lipid extract samples. The methyl esters of linolenic acid (18:3) and arachidonic acid (20:4) were eluted at the retention times of 20.9 and 22.3 min respectively (Fig 7). Presence of these two fatty acids were further confirmed by spiking the standards in the lipid extract and perform GC-SIM mode analysis after esterification, where these two fatty acids appeared at the same RTs as that of sample (Fig 7).

### Alpha-linolenic acid (ALA) triggers the growth of prostate cancer cell lines

**Fig 8:**
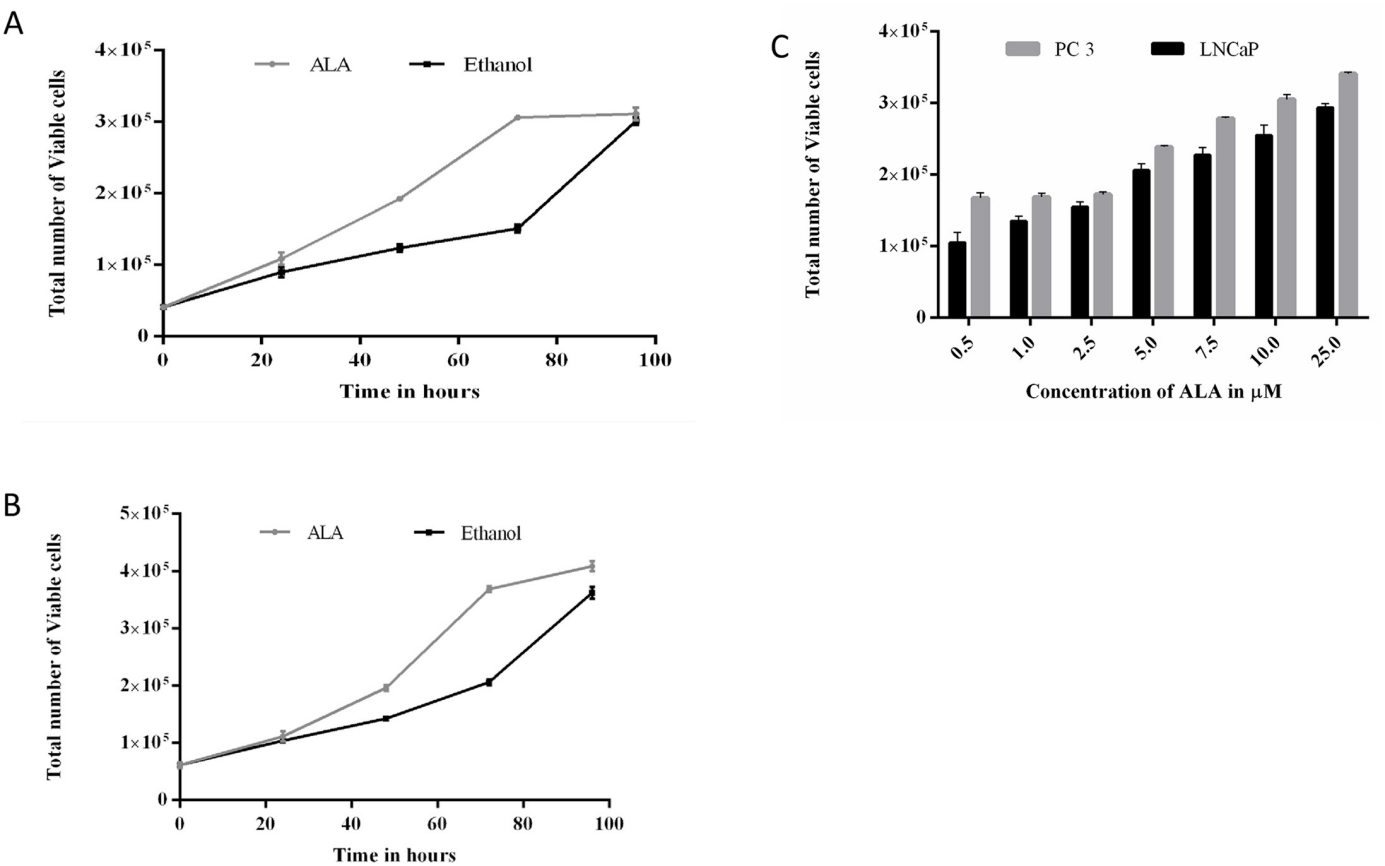
Effect of ALA on proliferation of LNCaP and PC-3 cells. (A&B) Proliferation of LNCaP and PC-3cells treated with different concentrations of ALA or Ethanol (solvent control) measured by using countess in cell viability analyzer (Invitrogen) over 72 h.(C&D) Time dependent effect of ALA on proliferation of LNCaP and PC-3 cells has been performed by measuring cell proliferation of ALA treated cells for 0 to 96 h. Initial number of cells seeded for assay was considered for 0 h.

To determine the effect of alpha-linolenic acid (18:3) on prostate cancer cell growth, we have treated the LNCaP and PC3 prostate cancer cells with alpha-linolenic acid both in dose and time dependent manner. In our investigations, we observed that alpha-linolenic acid induced growth of LNCaP cells significantly at lower concentrations even at 1 μM where as its effect on PC-3 cells growth was significant at higher concentrations. As presented in Fig 8A–8C, the dose and time dependent effect of ALA on the growth of both PCa cells is clearly suggesting its role in the proliferation of tumor cells. Further to determine the effect of ALA on morphology of proliferating PCa cells, the cells were imaged upon post treatment to ALA. The observed result showed that the cells are more proliferative and form clumps due to cell to cell interactions which could play an important role in increased cell proliferation up on ALA treatment (S2 Fig). Contrary to its pro proliferative activity, the concentration dependent effect of ALA on cancer cells demonstrate that the higher concentration of ALA at 100 μM inhibit cell proliferation thereby causing cell death (S3 Fig).

## Discussion

PCa is the most frequent type of cancer in men and is the second foremost cause of cancer deaths. The mortality rate of PCa has decreased dramatically with advent PSA test and DRE diagnostic methods. The PSA test is not prostate specific as a small amount of it is also detectable in other cancer types such as endometrial, breast, adrenal and renal cancers. As a result, routine screening of PCa patients with serum PSA test is still an open debate. Therefore, new biomarkers with a higher specificity and sensitivity may overcome limitations in PCa diagnosis. In this context, during the past decade a number of studies have performed genomic and proteomic profiling on radical prostatectomy tissues as well as extracellular fluids such as serum, plasma, urine and prostatic fluid [28–35]. These authors reported several potential markers that are less systemically studied for their sensitivity, specificity and accuracy in the diagnosis of PCa. Diagnosing cancer based on serum profiling is an attractive concept. Several studies have been performed protein and gene analysis on serum samples from cancer patients. Only sparse information is available for lipid composition in serum/plasma from cancer patients. However, research on lipids is still lacking compared to those of genes and proteins. Lipids are cellular metabolites and essential components of an organism. Lipids and lipid metabolism play an important role in many physiological functions involved in cellular signal transduction regulating cell proliferation, cell death and differentiation. However, profiling of disease related lipids specific to cancers is still lagging behind when compared to those of genes and proteins. This may be due to the very sparse information available on the functional role of lipids and/or limited technology for analyzing the complex lipids in biological specimens. With recent advances more attention has been drawn to blood serum/plasma lipids (lipidome) to investigate their role in cancer progression, diagnosis and cancer therapy. Lipids, including phospholipids and fatty acids have been found to be involved in PCa progression. In this study we focussed on lipidome analysis of serum samples collected from patients diagnosed with PCa and age matched healthy individuals as control. To avoid data variations due to loss of sample during extraction of lipids and normalize the instrument response, known amount of internal standard has been added to serum samples before extraction. Qualitative and quantitative comparison of lipid profiles between cancer and healthy individuals identify cancer-associated lipids that will be further evaluated for clinical relevance and to further understand their role in cancer progression. Extracellular fluids such as serum and plasma contain a variety of lipid species. A recent study described the composition of plasma lipids determined by lipidomics on pooled plasma samples [36]. The lipid species identified from our study overlapped with the list reported. Further normalization of apparent peak areas to internal standards identified differential lipid species between PCa and normal individuals.

Lipidomic data from the current study showed altered levels of24 lipids in serum from cancer patients compared to healthy controls. The list of identified lipids is heterogeneous belonging to different classes such as PC, PE, PA, FA, di and triglycerides. A recent report suggests that all phosphoglycerides, namely phosphatidylcholine, phosphatidylethanolamine, phosphatidylinositol were up regulated in PCa patients [7]. A three lipid signature including ePC 38:5, PC 40:3 and PC 42:4 is potential to screen patients for diagnosis of PCa [8]. However, in the present study, we have applied positive ion ESI, negative ion ESI and GC-MS approach to analyze different classes of lipids including fatty acids. Interestingly, we found that the fatty acids, tri and diglycerides are altered in PCa patient’s serum along with phospholipds. Other reports have shown that the plasma triglycerides are decreased in ovarian cancer whereas increased in breast cancer patients [37,38]. A higher TG level was associated with increased risk of breast cancer [37]. Similarly the serum triglyceride levels are high in colorectal carcinoma patientsand their risk is associated with elevated triglycerides in serum [39]. There is also evidence that more risk for PCa is positively associated with elevated TG levels in men over 60 years compared to younger males [40]. Differential phospholipid classes such as PA, PE and PC have been reported in breast cancer [41]. A case–cohort analysis study reported a positive association between plasma phospholipids, saturated fatty acids and PCa risk. But no significant association was observed to tumor aggressiveness [42]. Therefore, from the collected lipidomic data we sought to identify potential lipid signature with combination of different lipids which can classify tumor samples from healthy controls.

Hierarchical clustering of samples based on abundance of lipids led to clusters separating tumor specimen from normal. But four control samples became part of the tumor sample cluster. Interestingly, in two-dimensional clustering individual lipids identified did not lead to any specific cluster consisting same lipid species and/or class. Therefore, we were not able to identify a specific lipid class to consider as potential signature in distinguishing tumor from control specimens. To determine the effect of co-regulation of lipids on identification of biomarker, an unsupervised PCA has been applied to the abundance levels of altered lipids in cancer patients serum compared to corresponding controls. Here, as observed in cluster analysis, we could detect a clear separation of tumor with only one exception from normal patients and a separation of two distinct tumor groups based on the individual lipid abundance found in serum. Further data analysis with partition algorithms to find a lipid signature which can classify all samples with high certainty (distinguishing PCa patients and healthy controls) revealed more than one lipid are required to define sample identity. Among them serum lipid signatures identified in this study PC (39:6) and FA (22:3) together showed higher sensitivity, specificity and accuracy in differentiation of PCa specimens from normal compared to other lipids in possible combinations. Therefore, these lipids PC (39:6) and FA (22:3) may be possible serum biomarkers along with PSA test for diagnosis of PCa. Of course, further validation with large cohort of samples is needed to demonstrate sensitivity, specificity and accuracy of these lipids in classifying the tumors from normal.

Epidemiology reports have suggested an association of fatty acids with risk of prostate cancer [43]. From the studies presented so far it appears that some fatty acids are pro tumorigenic, promoting tumor growth and metastasis [44,45], whereas others show anti-tumor effect [46]. Particularly, fatty acids such as palmitic acid are known to play a key role in initiation and progression of PCa. Therefore, we made an attempt to assign representative peaks to fatty acids in GC-MS with known standards. Of all differential lipids, FA (18:3) was identified as α linolenic acid (ALA). High level of palmitic, myristic, linolenic, and eicosapentaenoic acids enhances the risk of prostate cancer and it has a inverse association with stearic acid [47]. Individual studies examining the association between ALA and PCa reported a positive association for the role of ALA in prostate carcinogenesis [48–51] except in one case which showed no association [52]. Reported data suggests that Arachidonic Acid, and linoleic acid (LA), an omega-6 polyunsaturated FA stimulates prostate cancer cell growth *in vitro* [53,54]. A recent study highlighted that the essential fatty acids promotes proliferation of both human PCa (PC3, LNCaP and TSU) cells as well as rat PCa (Mat-Ly-Lu (metastatic), EPYP2 and EPYP3 (non metastatic) cells[55]. Our results also showed an increase in proliferation of LNCaP and PC3 cells up on treatment with ALA in *in-vitro*. Interestingly, we observed that the higher concentration of ALA (50 and 100 μM) inhibiting proliferation of both LNCaP and PC3 cells contrary to the pro proliferative effect up to 25 μM. These observations are in agreement with the results reported by Pandalai et al. [55]. The lower concentration of fatty acids such as eicosapentanoic acid and arachidonic acid promotes growth of hormone dependent (LNCaP) and independent (PC3) PCa cells [53,56]. Contrarily, higher concentrations act negatively on proliferation of both cell types suggesting that the pro tumorigenic effect of essential fatty acids is concentration dependent [55,56]. However many mechanisms of action for fatty acids to different types of cancers progression have been hypothesized. ALA cannot be synthesized in humans. Therefore, serum levels of ALA are dependent on dietary intake only. The elevated levels of ALA in serum and prostate tissue may be due to altered ALA metabolism by desaturases [57,58]. Genetic variations particularly single nucleotide polymorphisms (SNPs) in gene encoded for delta-6-desaturase (*FADS2*) leading to higher linoleic acid (LA)/ALA ratio due to impaired metabolism [50]. In prostate cancer patients higher prostatic ALA is positively associated with serum PSA levels and cancer cell proliferation [59]. Fatty acids can regulate many cellular proteins in cancer cells directly or indirectly and there by influence DNA damage, tumor growth, angiogenesis and metastasis. The detailed mechanisms involved in ALA induced prostate carcinogenesis are worth pursuing.

## Conclusions

In conclusion, the current study using ESI-MS/MS reported serum lipids that are altered in PCa patients. Systematic analysis of the altered lipids helped us identify lipids PC 39:6 and FA 22:3distinguishes cancer patients from controls free from PCa. We also found that serum ALA levels are elevated in PCa. Moreover, LNCaP and PC-3 cells showed increased proliferation rate upon exposure to ALA in *in vitro*. Functional characterization of altered lipids will further substantiate their role in pathophysiology of prostate carcinogenesis. However, prospective clinical studies with large cohort of samples confirming diagnostic and prognostic potential of the lipid signatures are needed. With the results obtained from this study, we will continue to validate the identified signatures in a larger set of PCa and normal serum samples so that the findings will be useful in developing potential biomarker signatures.

## Supporting Information

S1 Fig GC-EIMS spectra of standard compounds of I.S (17:0) (a), (18:3) (b) and (20:4) (c) respectively. (TIF)

S2 Fig Effect of ALA on proliferation of LNCaP and PC3 cells. Both LNCaP and PC3 cells were treated with 25μM of ALA and cell morphology was observed after 24, 72 and 96 h of post treatment to determine effect of the ALA on proliferation of PCa cells.
(TIF)

S3 Fig Effect of ALA on proliferation of LNCaP and PC3 cells. The LNCaP and PC3 cells were treated with ALA of varying concentration (1 to 100 μM) for 48 h and cell morphology was observed to determine effect of the ALA on proliferation of PCa cells.
(TIF)

## Abbreviations

PCa: Prostate cancer
ALA: Alpha linolenic acid
ESI-MS: Electrospary Ionization Mass Spectrometry
GC-EIMS: Gas chromatography with electron ionization mass spectrometry

## References

[1] Jemal A, Siegel R, Xu J, Ward E (2010) Cancer statistics, 2010. CA Cancer J Clin 60(5): 277–300. 10.3322/caac.20073 20610543

[2] Scardino PT (1989) Early detection of prostate cancer. Urol Clin North Am 16(4): 635–655. 2479160

[3] Polascik TJ, Oesterling JE, Partin AW (1999) Prostate specific antigen: a decade of discovery—what we have learned and where we are going. J Urol 162(2): 293–306. 10411025 10.1016/s0022-5347(05)68543-6

[4] Watson AD (2006) Thematic review series: systems biology approaches to metabolic and cardiovascular disorders. Lipidomics: a global approach to lipid analysis in biological systems. J Lipid Res 47(10): 2101–2111. 16902246 10.1194/jlr.R600022-JLR200

[5] Gadomska H, Grzechocinska B, Janecki J, Nowicka G, Powolny M, Marianowski L et al (2005) Serum lipids concentration in women with benign and malignant ovarian tumours. Eur J Obstet Gynecol Reprod Biol 120(1): 87–90. 15866092 10.1016/j.ejogrb.2004.02.045

[6] Szachowicz-Petelska B, Sulkowski S, Figaszewski ZA (2007) Altered membrane free unsaturated fatty acid composition in human colorectal cancer tissue. Mol Cell Biochem 294(1–2): 237–242. 16858511 10.1007/s11010-006-9264-x

[7] Zhou X, Mao J, Ai J, Deng Y, Roth MR, Pound C et al (2012) Identification of plasma lipid biomarkers for prostate cancer by lipidomics and bioinformatics. PLoS One 7(11): e48889 10.1371/journal.pone.0048889 23152813 PMC3495963

[8] Patel N, Vogel R, Chandra-Kuntal K, Glasgow W, Kelavkar U (2014) A novel three serum phospholipid panel differentiates normal individuals from those with prostate cancer. PLoS One 9(3): e88841 10.1371/journal.pone.0088841 24603597 PMC3945968

[9] Pietilainen KH, Sysi-Aho M, Rissanen A, Seppanen-Laakso T, Yki-Jarvinen H, Kaprio J et al (2007) Acquired obesity is associated with changes in the serum lipidomic profile independent of genetic effects—a monozygotic twin study. PLoS One 2(2): e218 17299598 10.1371/journal.pone.0000218 PMC1789242

[10] Ekroos K, Janis M, Tarasov K, Hurme R, Laaksonen R (2010) Lipidomics: a tool for studies of atherosclerosis. Curr Atheroscler Rep 12(4): 273–281. 10.1007/s11883-010-0110-y 20425241 PMC2878593

[11] Graessler J, Schwudke D, Schwarz PE, Herzog R, Shevchenko A, Bornstein SR et al (2009) Top-down lipidomics reveals ether lipid deficiency in blood plasma of hypertensive patients. PLoS One 4(7): e6261 10.1371/journal.pone.0006261 19603071 PMC2705678

[12] Han X, Yang J, Yang K, Zhao Z, Abendschein DR, Gross RW (2007) Alterations in myocardial cardiolipin content and composition occur at the very earliest stages of diabetes: a shotgun lipidomics study. Biochemistry 46(21): 6417–6428. 17487985 10.1021/bi7004015 PMC2139909

[13] Ollero M, Astarita G, Guerrera IC, Sermet-Gaudelus I, Trudel S, Piomelli D et al (2011) Plasma lipidomics reveals potential prognostic signatures within a cohort of cystic fibrosis patients. J Lipid Res 52(5): 1011–1022. 10.1194/jlr.P013722 21335323 PMC3073467

[14] Gorke R, Meyer-Base A, Wagner D, He H, Emmett MR, Conrad CA (2010) Determining and interpreting correlations in lipidomic networks found in glioblastoma cells. BMC Syst Biol 4: 126 10.1186/1752-0509-4-126 20819237 PMC2944140

[15] BLIGH EG, DYER WJ (1959) A rapid method of total lipid extraction and purification. Can J Biochem Physiol 37(8): 911–917. 13671378 10.1139/o59-099

[16] Bird SS, Marur VR, Sniatynski MJ, Greenberg HK, Kristal BS (2011) Lipidomics profiling by high-resolution LC-MS and high-energy collisional dissociation fragmentation: focus on characterization of mitochondrial cardiolipins and monolysocardiolipins. Anal Chem 83(3): 940–949. 10.1021/ac102598u 21192696 PMC3031668

[17] Cequier-Sanchez E, Rodriguez C, Ravelo AG, Zarate R (2008) Dichloromethane as a solvent for lipid extraction and assessment of lipid classes and fatty acids from samples of different natures. J Agric Food Chem 56(12): 4297–4303. 10.1021/jf073471e 18505264

[18] Wishart DS, Jewison T, Guo AC, Wilson M, Knox C, Liu Y et al (2013) HMDB 3.0—The Human Metabolome Database in 2013. Nucleic Acids Res 41(Database issue): D801–D807. 10.1093/nar/gks1065 23161693 PMC3531200

[19] Applications of combinatorics and graph theory to the biological and social sciences In: Roberts F, ed, Conceptual scaling. NewYork: Springer, 1989: 139–167.

[20] Mehdi Kaytoue1 Se DSOKaAN (2009) Two FCA-Based Methods for Mining Gene Expression Data. Springer: 251–266.

[21] Motameny S, Versmold B, Schmutzler R (2008) Formal concept analysis for the identification of combinatorial biomarkers in breast cancer. In Proceedings of the 6th International Conference on Formal Concept Analysis: 229–240.

[22] Nilsson A, Fehniger TE, Gustavsson L, Andersson M, Kenne K, Marko Varga G et al (2010) Fine mapping the spatial distribution and concentration of unlabeled drugs within tissue micro-compartments using imaging mass spectrometry. PLoS One 5(7): e11411 10.1371/journal.pone.0011411 20644728 PMC2904372

[23] Brugger B, Erben G, Sandhoff R, Wieland FT, Lehmann WD (1997) Quantitative analysis of biological membrane lipids at the low picomole level by nano-electrospray ionization tandem mass spectrometry. Proc Natl Acad Sci U S A 94(6): 2339–2344. 9122196 10.1073/pnas.94.6.2339 PMC20089

[24] Liebisch G, Lieser B, Rathenberg J, Drobnik W, Schmitz G (2004) High-throughput quantification of phosphatidylcholine and sphingomyelin by electrospray ionization tandem mass spectrometry coupled with isotope correction algorithm. Biochim Biophys Acta 1686(1–2): 108–117. 15522827 10.1016/j.bbalip.2004.09.003

[25] Kerwin JL, Wiens AM, Ericsson LH (1996) Identification of fatty acids by electrospray mass spectrometry and tandem mass spectrometry. J Mass Spectrom 31(2): 184–192. 8799272 10.1002/(SICI)1096-9888(199602)31:2<184::AID-JMS283>3.0.CO;2-2

[26] Lukk M, Kapushesky M, Nikkila J, Parkinson H, Goncalves A, Huber W et al (2010) A global map of human gene expression. Nat Biotechnol 28(4): 322–324. 10.1038/nbt0410-322 20379172 PMC2974261

[27] Sanchez-Avila N, Mata-Granados JM, Ruiz-Jimenez J, Luque dC (2009) Fast, sensitive and highly discriminant gas chromatography-mass spectrometry method for profiling analysis of fatty acids in serum. J Chromatogr A 1216(40): 6864–6872. 10.1016/j.chroma.2009.08.045 19729166

[28] Dwivedi S, Goel A, Natu SM, Mandhani A, Khattri S, Pant KK (2011) Diagnostic and prognostic significance of prostate specific antigen and serum interleukin 18 and 10 in patients with locally advanced prostate cancer: a prospective study. Asian Pac J Cancer Prev 12(7): 1843–1848. 22126577

[29] Sreekumar A, Poisson LM, Rajendiran TM, Khan AP, Cao Q, Yu J et al (2009) Metabolomic profiles delineate potential role for sarcosine in prostate cancer progression. Nature 457(7231): 910–914. 10.1038/nature07762 19212411 PMC2724746

[30] Tomlins SA, Mehra R, Rhodes DR, Cao X, Wang L, Dhanasekaran SM et al (2007) Integrative molecular concept modeling of prostate cancer progression. Nat Genet 39(1): 41–51. 17173048 10.1038/ng1935

[31] Ummanni R, Mundt F, Pospisil H, Venz S, Scharf C, Barett C et al (2011) Identification of clinically relevant protein targets in prostate cancer with 2D-DIGE coupled mass spectrometry and systems biology network platform. PLoS One 6(2): e16833 10.1371/journal.pone.0016833 21347291 PMC3037937

[32] Pang J, Liu WP, Liu XP, Li LY, Fang YQ, Sun QP et al (2010) Profiling protein markers associated with lymph node metastasis in prostate cancer by DIGE-based proteomics analysis. J Proteome Res 9(1): 216–226. 10.1021/pr900953s 19894759

[33] Meehan KL, Holland JW, Dawkins HJ (2002) Proteomic analysis of normal and malignant prostate tissue to identify novel proteins lost in cancer. Prostate 50(1): 54–63. 11757036 10.1002/pros.10032

[34] Everley PA, Bakalarski CE, Elias JE, Waghorne CG, Beausoleil SA, Franzen B et al (2006) Enhanced analysis of metastatic prostate cancer using stable isotopes and high mass accuracy instrumentation. J Proteome Res 5(5): 1224–1231. 16674112 10.1021/pr0504891

[35] Lexander H, Palmberg C, Hellman U, Auer G, Hellstrom M et al (2006) Correlation of protein expression, Gleason score and DNA ploidy in prostate cancer. Proteomics 6(15): 4370–4380. 16888723 10.1002/pmic.200600148

[36] Han X, Gross RW (2003) Global analyses of cellular lipidomes directly from crude extracts of biological samples by ESI mass spectrometry: a bridge to lipidomics. J Lipid Res 44(6): 1071–1079. 12671038 10.1194/jlr.R300004-JLR200

[37] Franky DS, Shilin NS, Pankaj MS, Patel HR, Prabhudas SP (2008) Significance of alterations in plasma lipid profile levels in breast cancer. Integr Cancer Ther 7(1): 33–41. 10.1177/1534735407313883 18292593

[38] Qadir MI, Malik SA (2008) Plasma lipid profile in gynecologic cancers. Eur J Gynaecol Oncol 29(2): 158–161. 18459552

[39] Yamada K, Araki S, Tamura M, Sakai I, Takahashi Y, Kashihara H et al (1998) Relation of serum total cholesterol, serum triglycerides and fasting plasma glucose to colorectal carcinoma in situ. Int J Epidemiol 27(5): 794–798. 9839735 10.1093/ije/27.5.794

[40] Hayashi N, Matsushima M, Yamamoto T, Sasaki H, Takahashi H, Egawa S et al (2012) The impact of hypertriglyceridemia on prostate cancer development in patients aged >/ = 60 years. BJU Int 109(4): 515–519. 10.1111/j.1464-410X.2011.10358.x 21812901

[41] Sterin M, Cohen JS, Ringel I (2004) Hormone sensitivity is reflected in the phospholipid profiles of breast cancer cell lines. Breast Cancer Res Treat 87(1): 1–11. 15377845 10.1023/B:BREA.0000041572.07837.ec

[42] Bassett JK, Severi G, Hodge AM, MacInnis RJ, Gibson RA, Hopper JL et al (2013) Plasma phospholipid fatty acids, dietary fatty acids and prostate cancer risk. Int J Cancer 133(8): 1882–1891. 10.1002/ijc.28203 23575905

[43] Rose DP (1997) Effects of dietary fatty acids on breast and prostate cancers: evidence from in vitro experiments and animal studies. Am J Clin Nutr 66(6 Suppl): 1513S–1522S. 9394709 10.1093/ajcn/66.6.1513S

[44] Ghosh J, Myers CE Jr. (1998) Arachidonic acid metabolism and cancer of the prostate. Nutrition 14(1): 48–49. 9437681 10.1016/s0899-9007(97)00392-4

[45] De Vries CE, van Noorden CJ (1992) Effects of dietary fatty acid composition on tumor growth and metastasis. Anticancer Res 12(5): 1513–1522. 1444214

[46] Zhou JR, Blackburn GL (1997) Bridging animal and human studies: what are the missing segments in dietary fat and prostate cancer? Am J Clin Nutr 66(6 Suppl): 1572S–1580S. 9394717 10.1093/ajcn/66.6.1572S

[47] Crowe FL, Allen NE, Appleby PN, Overvad K, Aardestrup IV, Johnsen NF et al (2008) Fatty acid composition of plasma phospholipids and risk of prostate cancer in a case-control analysis nested within the European Prospective Investigation into Cancer and Nutrition. Am J Clin Nutr 88(5): 1353–1363. 18996872 10.3945/ajcn.2008.26369

[48] De SE, eo-Pellegrini H, Boffetta P, Ronco A, Mendilaharsu M (2000) Alpha-linolenic acid and risk of prostate cancer: a case-control study in Uruguay. Cancer Epidemiol Biomarkers Prev 9(3): 335–338. 10750674

[49] Giovannucci E, Rimm EB, Colditz GA, Stampfer MJ, Ascherio A, Chute CG et al (1993) A prospective study of dietary fat and risk of prostate cancer. J Natl Cancer Inst 85(19): 1571–1579. 8105097 10.1093/jnci/85.19.1571

[50] Gann PH, Hennekens CH, Sacks FM, Grodstein F, Giovannucci EL, Stampfer MJ et al (1994) Prospective study of plasma fatty acids and risk of prostate cancer. J Natl Cancer Inst 86(4): 281–286. 8158682 10.1093/jnci/86.4.281

[51] Harvei S, Bjerve KS, Tretli S, Jellum E, Robsahm TE, Vatten L (1997) Prediagnostic level of fatty acids in serum phospholipids: omega-3 and omega-6 fatty acids and the risk of prostate cancer. Int J Cancer 71(4): 545–551. 9178806 10.1002/(sici)1097-0215(19970516)71:4<545::aid-ijc7>3.0.co;2-u

[52] Andersson SO, Wolk A, Bergstrom R, Giovannucci E, Lindgren C et al (1996) Energy, nutrient intake and prostate cancer risk: a population-based case-control study in Sweden. Int J Cancer 68(6): 716–722. 8980172 10.1002/(SICI)1097-0215(19961211)68:6<716::AID-IJC4>3.0.CO;2-6

[53] Ghosh J, Myers CE (1997) Arachidonic acid stimulates prostate cancer cell growth: critical role of 5-lipoxygenase. Biochem Biophys Res Commun 235(2): 418–423. 9199209 10.1006/bbrc.1997.6799

[54] Rose DP, Connolly JM (1991) Effects of fatty acids and eicosanoid synthesis inhibitors on the growth of two human prostate cancer cell lines. Prostate 18(3): 243–254. 2020620 10.1002/pros.2990180306

[55] Pandalai PK, Pilat MJ, Yamazaki K, Naik H, Pienta KJ (1996) The effects of omega-3 and omega-6 fatty acids on in vitro prostate cancer growth. Anticancer Res 16(2): 815–820. 8687134

[56] Aronson WJ, Kobayashi N, Barnard RJ, Henning S, Huang M, Jardack PM et al (2011) Phase II prospective randomized trial of a low-fat diet with fish oil supplementation in men undergoing radical prostatectomy. Cancer Prev Res (Phila) 4(12): 2062–2071. 22027686 10.1158/1940-6207.CAPR-11-0298 PMC3232341

[57] Goyens PL, Spilker ME, Zock PL, Katan MB, Mensink RP (2006) Conversion of alpha-linolenic acid in humans is influenced by the absolute amounts of alpha-linolenic acid and linoleic acid in the diet and not by their ratio. Am J Clin Nutr 84(1): 44–53. 16825680 10.1093/ajcn/84.1.44

[58] Cho HP, Nakamura MT, Clarke SD (1999) Cloning, expression, and nutritional regulation of the mammalian Delta-6 desaturase. J Biol Chem 274(1): 471–477. 9867867 10.1074/jbc.274.1.471

[59] Azrad M, Zhang K, Vollmer RT, Madden J, Polascik TJ, Snyder DC et al (2012) Prostatic alpha-linolenic acid (ALA) is positively associated with aggressive prostate cancer: a relationship which may depend on genetic variation in ALA metabolism. PLoS One 7(12): e53104 10.1371/journal.pone.0053104 23285256 PMC3532426

