# Supplementary figures and images for: Prostate Cancer Associated Lipid Signatures in Serum Studied by ESI-Tandem Mass Spectrometryas Potential New Biomarkers
Source: PLoS One. 2016 Mar 9;11(3):e0150253. doi: 10.1371/journal.pone.0150253 (PMC4784901; doi:10.1371/journal.pone.0150253)

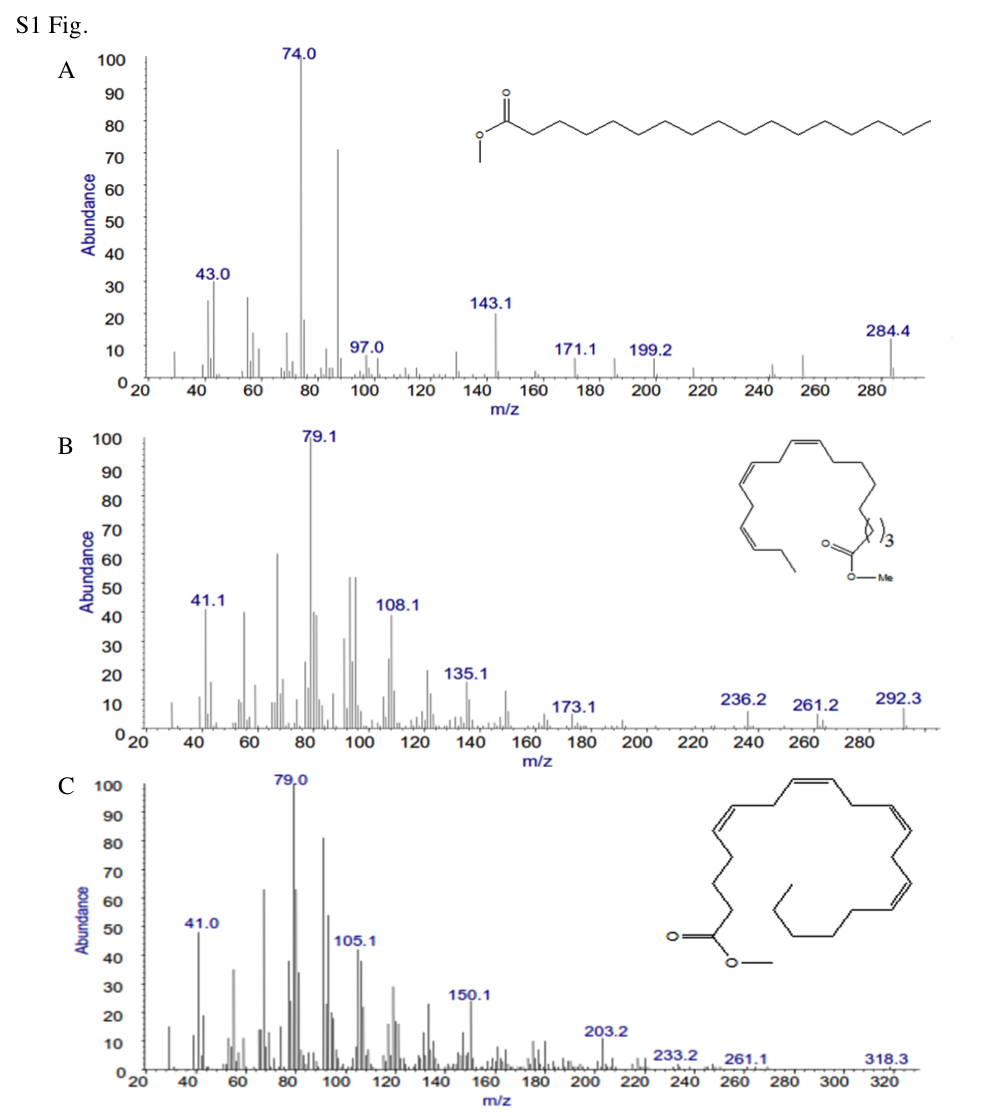

Supplement: S1 Fig — (TIF) [file pone.0150253.s001.tif]

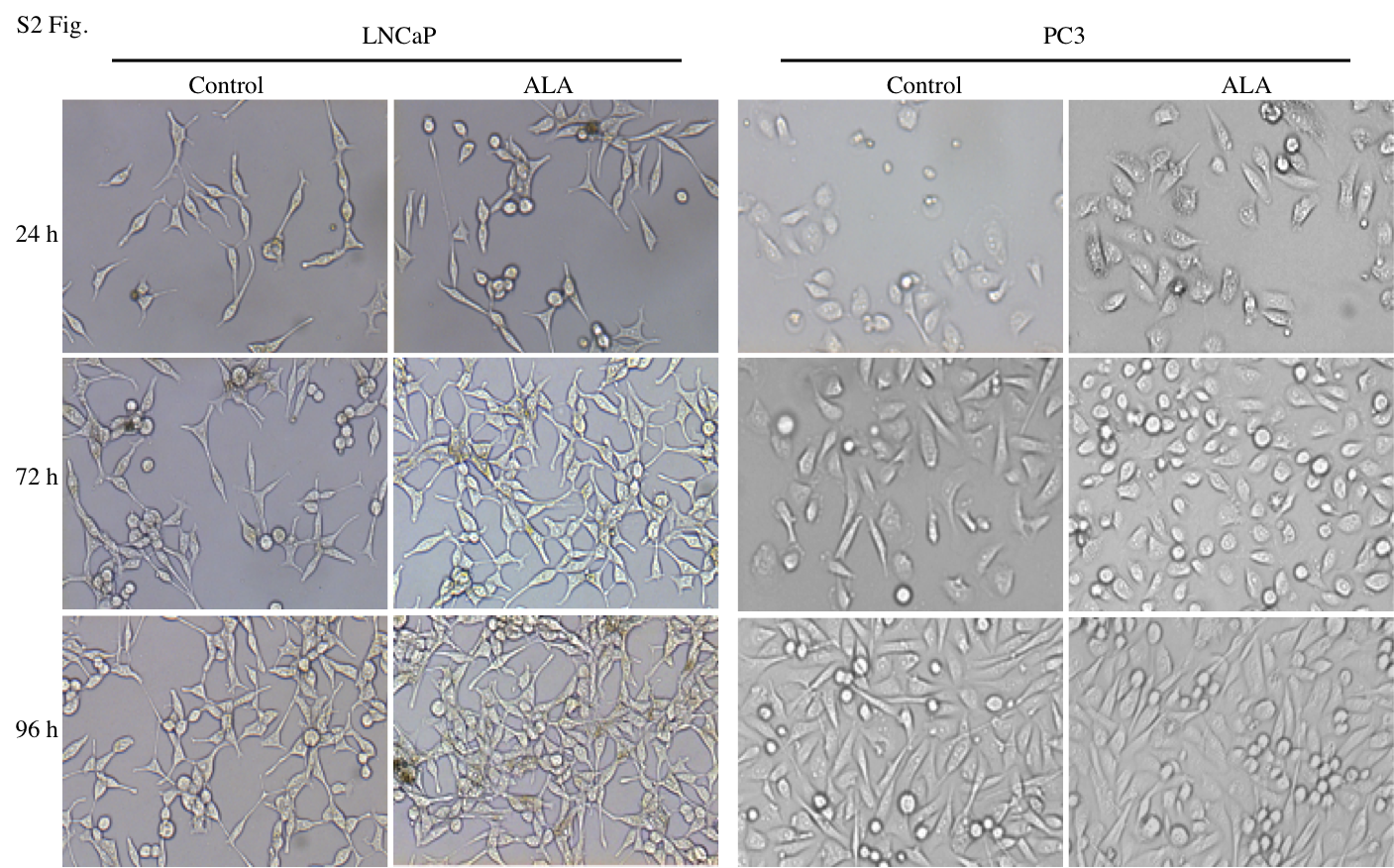

Supplement: S2 Fig — Both LNCaP and PC3 cells were treated with 25μM of ALA and cell morphology was observed after 24, 72 and 96 h of post treatment to determine effect of the ALA on proliferation of PCa cells. (TIF) [file pone.0150253.s002.tif]

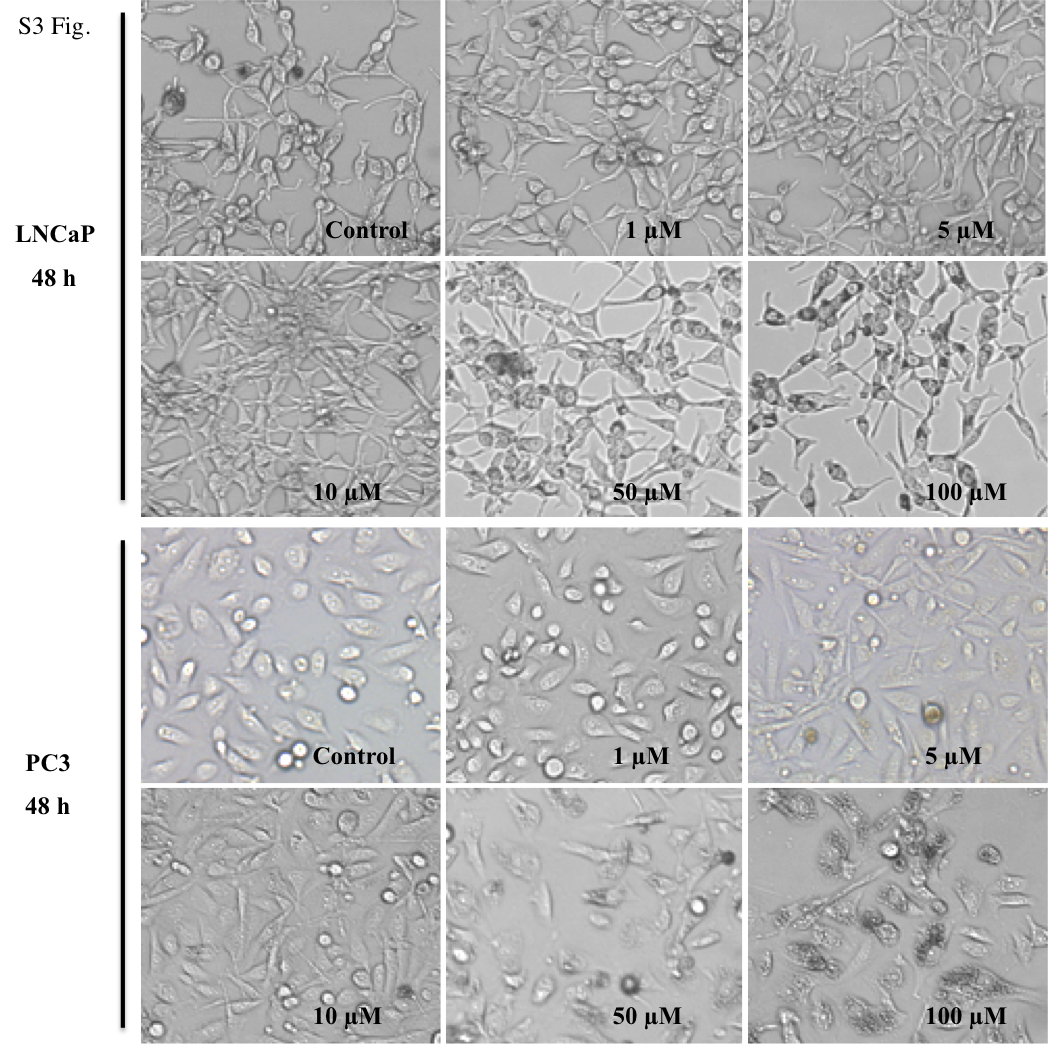

Supplement: S3 Fig — The LNCaP and PC3 cells were treated with ALA of varying concentration (1 to 100 μM) for 48 h and cell morphology was observed to determine effect of the ALA on proliferation of PCa cells. (TIF) [file pone.0150253.s003.tif]
